# Bolton ratios in Portuguese subjects among different malocclusion groups

**DOI:** 10.4317/jced.54977

**Published:** 2018-09-01

**Authors:** Vanessa Machado, João Botelho, Dinis Pereira, Mariana Vasques, Paulo Fernandes-Retto, Luís Proença, José-João Mendes, Ana Delgado

**Affiliations:** 1 Clinical Research Unit (CRU), Centro de Investigação Interdisciplinar Egas Moniz (CiiEM), Egas Moniz Cooperativa de Ensino Superior, C.R.L., Monte de Caparica, Almada, Portugal; 2 Egas Moniz Cooperativa de Ensino Superior, C.R.L., Monte de Caparica, Almada, Portugal

## Abstract

**Background:**

Several methods have been described to estimate inter-arch tooth size relationship, such as Bolton’s ratios. The aims of this study were to verify the validity of Bolton indexes in a sample of untreated Portuguese subjects based on Angle classification and to evaluate the gender difference.

**Material and Methods:**

168 pre-treatment dental casts of orthodontics Portuguese subjects (59 males and 109 females) with different occlusions were used, which were selected randomly from 541 consecutively treated orthodontic patients. The mesiodistal widths from first molar to first molar were measured on each pre-treatment cast to the nearest 0.01 mm using digital caliper, and Bolton’s anterior and overall ratios were calculated. Descriptive statistics as mean, standard deviation and range were calculated. Moreover, the results were compared to Bolton’s ratios and differences based on gender and occlusion groups were evaluated by statistical inference methods.

**Results:**

The results reveal that the mean values, standard deviation and range were larger than Bolton’s in normal occlusion group (78.3±3.5% in anterior ratio and 92.1±2.2% in overall ratio) and there were no differences between genders (*p* >0.05). Class I (anterior and overall ratios, *p*=0.001 and *p*<0.001, respectively), Class II/2 (anterior ratio, *p*=0.032) and Class III (overall ratio, *p*=0.041) were significantly different from Bolton’s reference data.

**Conclusions:**

The results showed no differences between gender and no difference between normal occlusion and malocclusion groups. Moreover, in normal occlusion group, the anterior and overall tooth size ratios was equivalent to the original Bolton’s ratios, although the mean and standard deviation were large.

** Key words:**Bolton anterior and overall ratios, normal occlusion, malocclusion, portuguese population.

## Introduction

Human dentition is one of the most complex adaptive system, and is influenced by genetic, epigenetic, and environmental factors, having an anthropological significance (1–6). Besides that, sexual dimorphism of the teeth dimension is related to humanoid sex genes and hormones and is influenced by their imbalance (7–9). Furthermore, teeth size have been studied worldwide in order to compare various populations that have specific characteristics and to determine the patterns of variability between different teeth, associations within and between dental arches (1,2,5,6,9–15).

Ideally, the patient needs a perfect inter-arch relationship with normal overjet and overbite, to have the attainment of a normal occlusion. For this, it requires the existence of proportional maxillary and mandibular teeth. In some cases, patients have significant tooth size discrepancy (TSD) and orthodontic alignment into ideal occlusion may not be possible per se (16,17).

Several methods have been described to estimate inter-arch tooth size relationship, such as Bolton’s ratios. These allow the orthodontist to obtain information about the maxillary-to-mandibular tooth size relationship (6,18). Bolton established ideal anterior and overall ratios with mean values of 77.2% and 91.3%, respectively (16,17). Nevertheless, in diagnosis and treatment planning, Bolton’s ratios should not be generalized for all patients since the proposed standard values are questionable (12).

The aims of the present study were verify the validity of the Bolton indexes in a sample of untreated Portuguese subjects based on Angle classification and to compare the gender difference.

## Material and Methods

-Ethics

This study was approved by Egas Moniz Ethics Committee (process 600) and was carried out in accordance with the Helsinki Declaration of 1975 as revised in 2013. A written informed consent was obtained from all participants during the first orthodontic appointment. All data were registered on a database specifically created for this purpose, where coded number was attributed to each participant. This was a retrospective observational study without study-defined medical or dental interventions.

-Patient selection 

The assessment tool consisted of pre-treatment dental casts of patients seeking for orthodontic treatment, selected from the archives of the Orthodontic Department, from Egas Moniz Dental Clinic (Almada, Portugal). From a total of 541 pre-treatment casts gathered from November 2010 to December 2017, 168 (59 males and 109 females) were randomly selected according to the inclusion and exclusion criteria.

The inclusion criteria were: all teeth were fully erupted and present, from first molar in the right side to first molar in the left side in both upper and lower jaws, no history of extraction or interproximal stripping, no proximal caries that might interfere with precise tooth measurement, restorations, abrasion or attrition, no previous or ongoing orthodontic treatment, no abnormal tooth morphology and congenitally missing impacted and grossly carious teeth.

-Angle classification/anteroposterior relationship 

The Angle classification was used to divide patients according to the type of occlusion classification. The anteroposterior relationship was established from the relation of the first maxillary and first mandibular molars and the canines, bilaterally.

After scrutinizing the samples based on inclusion and exclusion criteria, the final sample consisted of 168 models with 29 patients having normal occlusion, 50 with Class I malocclusion, 23 with Class II division 1, 28 with Class II division 2 and 38 with Class III.

-Dental casts analysis

All dental casts measurements and analysis were performed by the same person (VM) using digital caliper to measure the mesiodistal tooth widths from the right first molar to the left first molar, to the nearest 0.01 mm. The mesiodistal width of each tooth was measured at the widest distance between the mesial and distal contact points. The position of the caliper had to be perpendicular to the occlusal surface of the measured tooth. Anterior and overall ratios were calculated for each sample using the formula as proposed by Bolton (11).

-Measurement reproducibility 

In order to assess the error of the method, 10 study casts were randomly chosen from the total of 168 and remeasured one week later by the same investigator. Intra-class correlation coefficients were calculated with absolute agreement of 0.94 and 0.92 for overall and anterior ratios, respectively.

-Statistical Analysis

Data analysis was performed using IBM SPSS Statistics version 24.0 for Windows (Armonk, NY: IBM Corp.). Descriptive statistics as mean and standard deviation were calculated for the size of the teeth. Population means were estimated by calculating 95% confidence intervals (95% CI). In the comparative statistical inference analysis, the level of significance was set at 5%.

## Results

In this Portuguese sample (mean age 20.1 ± 7.3), both male and female measurements follow a similar pattern distribution, but the difference in males and females was clearly evident.

 Table 1 and  Table 2 summarizes the mean values, standard deviation, range and 95% confidence intervals for means of the anterior and overall tooth size ratios in normal occlusion and malocclusion groups, for both genders. As shown, no statistically significant differences were found between males and females mean values, in either anterior or overall ratio, for normal occlusion (*p*= 0.320 and *p*= 0.165, respectively) or among the malocclusion classes (*p*> 0.05). Therefore, males and females values were fully assessed for all subsequent analyses (Table For the anterior ratio, this Portuguese sample had a mean of 78.3 (±3.5)% in the normal occlusion group (95% CI 76.9-79.6%) ( Table 1). For the overall ratio, the mean was 92.1 (±2.2)%, (95% CI 91.3-92.9%), for the normal occlusion group ( Table 2). No statistically significant differences were found in anterior and overall ratios between the normal occlusion and malocclusion groups (*p*=0.693 and *p*=0.214, ANOVA, respectively).

**Table 1:**
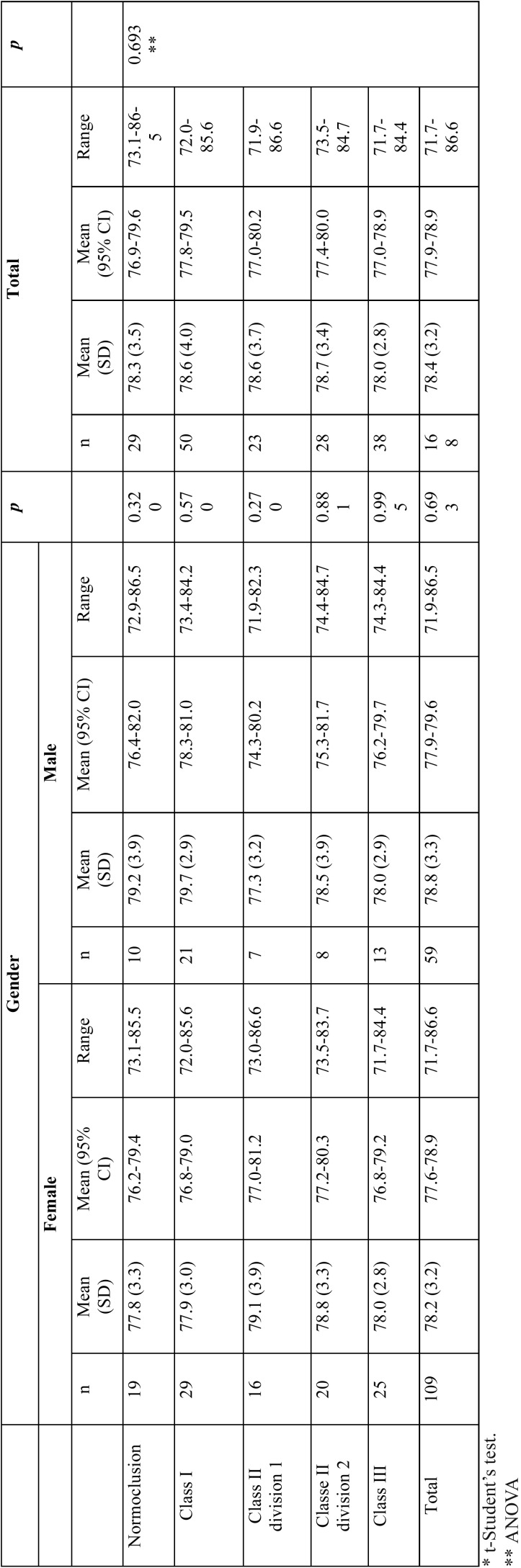
Anterior ratio (%) of tooth size discrepancy (mean, standard deviation, range and 95% CI for mean). Gender comparison for normal occlusion and malocclusion groups.

**Table 2:**
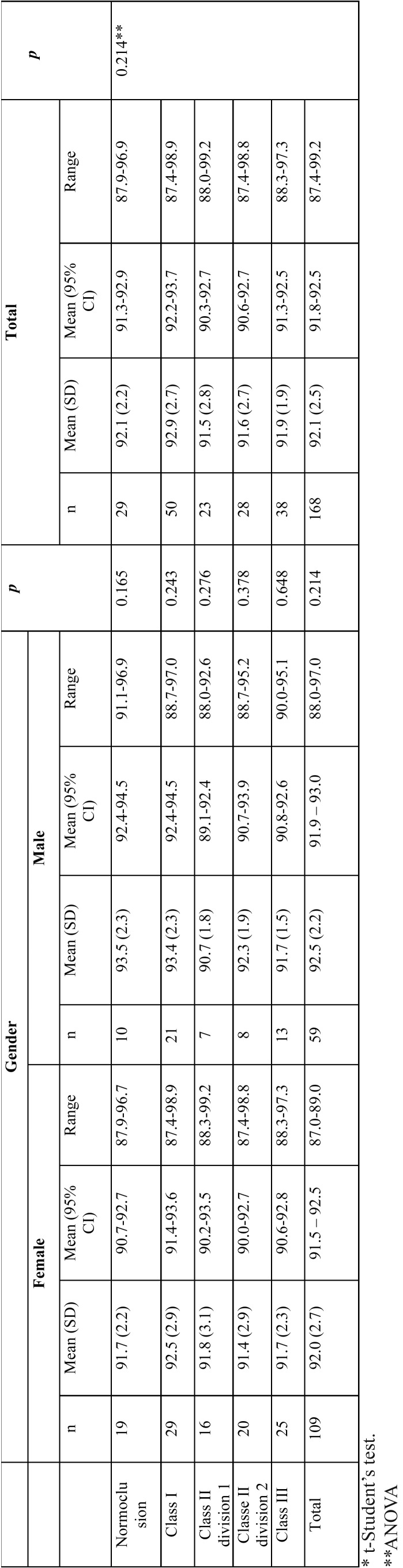
Overall ratio (%) of tooth size discrepancy (mean, standard deviation, range and 95% CI for mean). Gender comparison for normal occlusion and malocclusion groups.

In  Table 3, when comparing the anterior ratios with Bolton’s value, Class I and Class II/2 showed statistically significant differences (*p*=0.001 and *p*=0.032, one-sample t-Student’s test, respectively). When considering the overall ratio, Class I and Class III exhibited statistically significant differences when compared to Bolton’s reference values (*p*<0.001 and *p*=0.041, one-sample t-Student’s test, respectively). Moreover, in normal occlusion group, the anterior and overall tooth size ratios was equivalent to the original Bolton’s ratios (*p*=0.078 and *p*=0.055, respectively), although the mean and standard deviation were large.

**Table 3:**
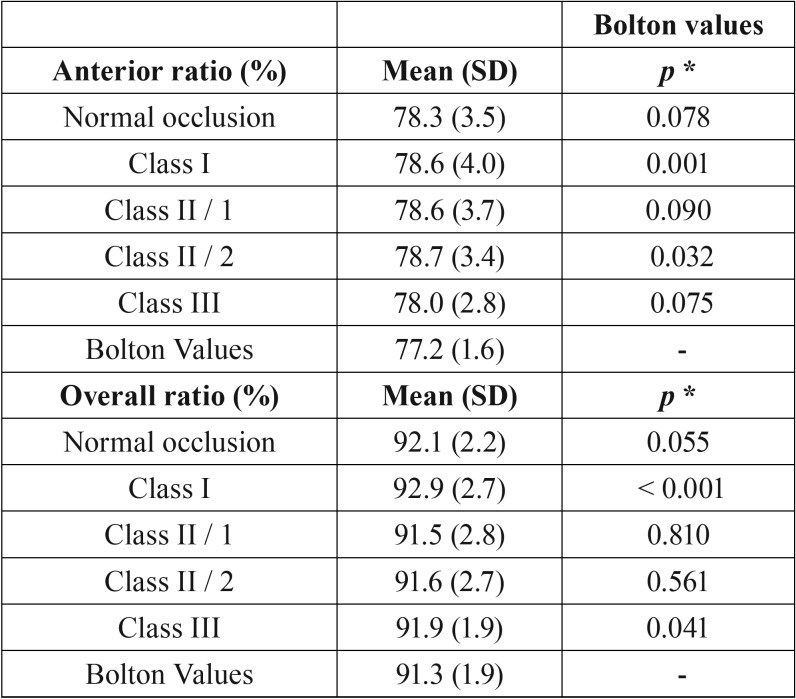
Comparison of anterior and overall ratio (%) of tooth size discrepancy with Bolton values.

## Discussion

The purpose of this study was to verify the validity of Bolton indexes (11) in an untreated Portuguese sample subjects based on Angle classification and to find if there were gender differences. The main finding was that the mean values, standard deviation and range were larger than Bolton’s values and there were no differences between genders.

It is commonly accepted that orthodontic treatment is based on careful diagnosis, comprehensive treatment planning and a correct interpretation of all findings. Moreover, identification of tooth size discrepancy is one of the key aspects to predict treatment outcomes, achieving balanced occlusion and obtain stable inter-digitation.

According to the present study, no statistically significant differences were found between genders in anterior or overall Bolton’s ratio for each malocclusion group. These findings are in the line with previous researches on several other populations (13–15,19–24). On the other hand, some other investigations showed statistically significant differences between genders in the overall ratio for the malocclusion groups (23,25) and in the anterior ratio (23,26). Therefore, it is speculated that gender differences in tooth size ratios may be population specific.

Nevertheless, studies assert that Bolton index can be correlated with the type of malocclusion (20,27). For example, Nie and Lin (28) measured this in 300 pre-orthodontic subjects according to their Angle malocclusion group. Contrary to expectations, Class III malocclusion group have the smallest mean and standard deviation in anterior and overall ratios when compared with the other malocclusion and normal occlusion groups. However, no statistically significant differences were observed in the mean overall and anterior ratios between Class I, Class II/1, Class II/2, Class III and normal occlusion. This finding is in agreement with those of previous studies on other populations (13,22,24,29).

When comparing the mean values of anterior and overall ratios of normal occlusion and malocclusion groups with Bolton’s reference values, there were no significant differences, except for Class I in both ratios, Class II/2 in the anterior ratio and Class III in the overall ratio. These results corroborate the findings of Crosby and Alexander (29), Paredes *et al.* (21) and Cançado *et al.* (22).

Furthermore, when we compared the mean and standard deviation of each group to Bolton’s values, all groups had greater deviation than Bolton’s. These findings can be explained by the inherent differences in the population of the two investigations. Bolton used a small and possible homogenous group, whereas this study was conducted in a learning institution that included subjects who seeking orthodontic treatment.

Bolton’s ratio remain the most recognized methods for detecting inter-arch tooth size discrepancies and gained wide acceptance in clinical orthodontics. Bolton studied 55 models classified as excellent occlusion, although 44 have been previously orthodontically treated (11). Also, population and gender composition of Bolton’s sample were not specified, which implies potential selection bias (23).

Furthermore, Bolton’s ratios have been developed to determine the necessity to reduce tooth width through interproximal stripping (17). However, Bolton didn’t consider microdontia which, in some cases, leads to the need to increase tooth mesiodistal size using prosthetic procedures. Besides that, since tooth widths depends on multiple factors, the generalized application of Bolton’s ratios and the proposed values for a harmonious dentition are questionable and may be invalid for other populations than Bolton’s.

## Conclusions

On the basis of the results of this investigation, the following conclusions can be drawn:

● There are no statistically differences between genders for normal occlusion or malocclusion groups.

● Mean values, standard deviation and range were larger than original Bolton’s.

● There were no statistically significant differences among the Class I; Class II, division 1; Class II, division 2; and Class III malocclusion groups for the anterior and overall ratio;

● In normal occlusion group, the anterior and overall tooth size ratios were equivalent to the original Bolton’s ratios, although mean and standard deviation were large.

● Anterior and overall ratios were significantly different from Bolton’s when considering Class I (anterior and overall ratios), Class II/2 (anterior ratio) and Class III (overall ratio) groups.
